# Corneal characteristics in Down syndrome patients with normal and keratoconic cornea

**DOI:** 10.3389/fmed.2022.985928

**Published:** 2022-09-16

**Authors:** Hassan Hashemi, Soheila Asgari

**Affiliations:** Noor Ophthalmology Research Center, Noor Eye Hospital, Tehran, Iran

**Keywords:** Down syndrome, corneal structure, reference range, keratoconic cornea, corneal tomography

## Abstract

**Purpose:**

To determine the reference range of corneal indices in Down syndrome patients with normal corneas (DS-N) and to compare it with the corneal indices in Down syndrome patients with keratoconic corneas (DS-KC).

**Methods:**

A study was conducted using the data of 154 eyes of 154 DS-N and 25 eyes of 25 DS-KC patients. Eighteen indices related to thickness, anterior chamber, keratometry, elevation, and aberrations routinely used for KC diagnosis were extracted from the Pentacam.

**Results:**

The mean age of the participants in DS-N and DS-KC groups was 16.73 ± 4.70 and 16.56 ± 4.22 years (*P* = 0.852). In the DS-N group, 95% CI were 511.65–520.31 for minimum corneal thickness, 2.97–3.07 for anterior chamber depth (ACD), 46.83–47.37 for maximum keratometry (Kmax), 46.13–46.62 for zonal Kmax at 3 mm, 0.35–0.58 for inferior-superior asymmetry (I-S value), 1.56–1.88 for Belin/Ambrósio display-total deviation, 8.65–10.79 for best-fit-sphere posterior elevation at the thinnest point, and 0.18–0.22 for corneal vertical coma. The age-related change in I-S value and corneal spherical aberration (SA) was significant (both *P* < 0.05). There were significant inter-gender differences in 11 indices; the female DS patients had shallower, steeper, more elevated, and more aberrated corneas (all *P* < 0.05). There were significant differences in all indices except for ACD (*P* = 0.372) and corneal SA (*P* = 0.169) between DS-N and DS-KC groups.

**Conclusion:**

In DS patients aged 10–30 years, the reference ranges of corneal indices are different from the range reported for non-DS subjects and are close to values reported for mild KC non-DS cases. The normal values are different between DS male and female; hence, sex-specific ranges should be considered for diagnosis of corneal abnormality in DS patients.

## Introduction

The prevalence of Down syndrome (DS) (1 in 700 babies born) (1), the high incidence of keratoconus (KC) (2) and KC compatible manifestations (3) in these patients underline the importance of determining the reference range of corneal indices for KC diagnosis. Several studies found distinct corneal structure differences between DS and non-DS populations; therefore, the reference range of the non-DS population cannot be applied to them. We previously analyzed and reported corneal indices including corneal thickness and volume from the center to the periphery (4), anterior chamber dimensions and angles (5) different types of keratometry (6) and corneal density (7) in a large sample of DS patients. Since the mean values of these indices were reported scatteredly in previous papers, this report was prepared to present the distribution of the main indices used for KC diagnosis in DS patients with normal (DS-N) and keratoconic (DS-KC) corneas in an organized manner. For this reason, in addition to mean and standard deviation (SD), 95% confidence interval (95%CI) and median were reported according to age and sex.

## Materials and methods

This research was conducted using the data of DS patients in Noor Eye Hospital, Tehran, Iran. The methodology of this study was reported in detail elsewhere (8). In brief, of 250 patients recruited from different sources, 225 patients aged 10–30 years were included in the study and assigned to three groups of DS with normal cornea (DS-N), DS with keratoconus suspect cornea (DS-KCS), and DS with keratoconic cornea (DS-KC). In DS-KC cases, stage of KC was II to IV using system of topographical keratoconus classification in Pentacam HR (Oculus Optikgeräte GmbH, Wetzlar, Germany). The diagnostic method and the clinical and paraclinical criteria were elaborated in another report (2). Briefly, KC diagnosis was made by two independent expert examiners using clinical findings (Vogt's striae, Munson's sign, apical thinning, Fleischer's ring, or Rizutti's sign) and abnormal topographic indices [maximum keratometry in a 3 mm zone around the steepest point (ZKmax-3 mm), maximum Ambrósio's relational thickness (ART-max), inferior-superior asymmetry (I-S value), Belin/Ambrosio display-total deviation (BAD-D), minimum corneal thickness (MCT), and the posterior elevation map)] measured by Pentacam HR. Only DS-N and DS-KC cases were evaluated in this report. Cases with a history of ocular surgery were not included in the study to determine the normal range of corneal indices without any bias.

The Ethics Committee of Tehran University of Medical Sciences (ID: 1397.091) approved the study and written informed consent was obtained from parents. However, if a child was uncooperative, the examinations were postponed to another time; in other words, verbal assent was obtained from each participant before any procedure.

In addition to ophthalmic examinations, visual acuity and refraction measurement, all subjects underwent imaging using the Pentacam HR between 8-12 am. Imaging continued until quality specification were OK; if this quality was not achieved after three attempts, the examination was rescheduled for the next 2–3 days.

The Pentacam data was extracted using the data management software (version: 1.21r43) and the following indices were used: apical corneal thickness (ACT), MCT, ART-max, anterior chamber depth (ACD), anterior chamber volume (ACV), maximum keratometry in the central 3 mm (Ksteep), minimum keratometry in the central 3 mm (Kflat), maximum keratometry in the central 8 mm (Kmax), ZKmax-3 mm, I-S value, anterior radius of curvature centered on the thinnest point (ARC), posterior radius of curvature centered on the thinnest point (PRC), BAD-D, 8.00 mm best-fit-sphere anterior elevation at the thinnest point (AE-Thin), 8.00 mm best-fit-sphere posterior elevation at the thinnest point (PE-Thin), corneal vertical coma, corneal spherical aberrations (SA), corneal higher order aberrations (HOA).

Statistical analysis was done using the STATA statistical software: release 14 (Stata, Corp. LP, College Station, TX, USA). Only the right eyes of the participants were analyzed to eliminate the correlation of fellow eyes. Normal distribution of indices was evaluated by Kolmogorov–Smirnov test and Q-Q plot. In addition to mean ± standard deviation (SD), 95% confidence interval (95% CI) and median were also used to present the distribution of the indices. A linear regression model was used to compare the indices between the two groups and two gender. The level of significance was set at 0.05.

## Results

In this study, 154 eyes of 154 DS-N (females: 55.2%) and 25 eyes of 25 DS-KC patients (females: 44%) with a mean age of 16.73 ± 4.70 and 16.56 ± 4.22 years were enrolled in the study (*P* = 0.852).

Table 1 presents the comparison of the indices between the two groups. Among tomographic indices, ACT (*P* = 0.034), MCT (*P* = 0.001), ART-max (*P* < 0.001), ACV (*P* = 0.036), Ksteep (*P* = 0.010), Kflat (*P* = 0.014), Kmax (*P* = 0.013), ZKmax-3 mm (*P* = 0.008), I-S value (*P* = 0.004), ARC (*P* = 0.002), PRC (*P* = 0.002), BAD-D (*P* = 0.009), AE-Thin (*P* = 0.014), PE-Thin (0.023), corneal vertical come (*P* = 0.005), and corneal HOAs (*P* = 0.008) showed significant differences between the two groups. However, no significant difference was found in ACD (*P* = 0.372) and corneal SA (*P* = 0.169) between them.

**Table 1:** Distribution of corneal indices in Down syndrome patients with normal cornea/DS-N (*n* = 154) and Down syndrome patients with keratoconic cornea/DS-KC (*n* = 25).

	**DS-N**			**DS-KC**		
	**Mean ±SD**	**95% CI**	**Median**	**Mean ±SD**	**95% CI**	**Median**
**ACT (μm)**	524.04 ± 29.80	519.30 to 528.79	521.00	506.04 ± 38.72	490.05 to 522.02	502.00
**MCT (μm)**	515.48 ± 30.36	511.65 to 520.31	514.00	492.28 ± 37.57	476.77 to 507.79	489.00
**ART-max (μm)**	408.51 ± 105.23	391.64 to 425.37	401.00	320.00 ± 113.20	273.27 to 366.73	316.00
ACD (mm)	3.02 ± 0.29	2.97 to 3.07	3.00	2.96 ± 0.27	2.85 to 3.07	2.99
**ACV (mm** ^**3**^ **)**	168.57 ± 31.15	163.61 to 173.52	165.00	154.28 ± 32.96	140.67 to 167.88	147.00
**Ksteep (D)**	46.52 ± 1.66	46.25 to 46.78	44.70	48.64 ± 3.72	47.10 to 50.17	46.40
**Kflat (D)**	44.79 ± 1.47	44.56 to 45.02	46.50	46.61 ± 3.40	45.21 to 48.02	48.60
**Kmax (D)**	47.10 ± 1.70	46.83 to 47.37	47.00	50.26 ± 5.88	47.83 to 52.68	49.47
**ZKmax-3mm (D)**	46.38 ± 1.55	46.13 to 46.62	46.47	49.13 ± 4.77	47.16 to 51.10	48.34
**I-S value (D)**	0.46 ± 0.73	0.35 to 0.58	0.50	1.98 ± 2.34	1.01 to 2.94	1.65
**ARC (mm)**	7.39 ± 0.24	7.36 to 7.43	7.37	7.04 ± 0.50	6.83 to 7.24	7.07
**PRC (mm)**	6.10 ± 0.25	6.06 to 6.14	6.10	5.72 ± 0.55	5.49 to 5.94	5.73
**BAD-D**	1.72 ± 1.02	1.56 to 1.88	1.65	3.55 ± 3.21	2.23 to 4.88	2.83
**AE-Thin (μm)**	4.19 ± 2.97	3.71 to 4.67	4.00	9.16 ± 9.29	5.33 to 12.99	7.00
**PE-Thin (μm)**	9.72 ± 6.67	8.65 to 10.79	9.00	19.32 ± 19.61	11.22 to 27.42	15.00
**Total vertical coma (μm)**	0.20 ± 0.13	0.18 to 0.22	0.18	0.66 ± 0.70	0.35 to 0.96	0.49
Total SA (μm)	0.16 ± 0.09	0.15 to 0.18	0.16	0.21 ± 0.34	0.06 to 0.36	0.12
**Total HOA (μm)**	0.57 ± 0.18	0.54 to 0.60	0.55	1.12 ± 0.89	0.73 to 1.50	0.86

Bold notations: significant differences between DS-N and DS-KC.

Apical corneal thickness (ACT), minimum corneal thickness (MCT), maximum Ambrósio's relational thickness (ART-max), anterior chamber depth (ACD), anterior chamber volume(ACV), maximum keratometry in the central 3 mm (Ksteep), minimum keratometry in the central 3 mm (Kflat), maximum keratometry in the central 8 mm (Kmax), maximum keratometry in a 3 mm zone around the steepest point (ZKmax-3mm), inferior-superior dioptric asymmetry (I-S value), anterior radius of curvature centered on the thinnest point (ARC), posterior radius of curvature centered on the thinnest point (PRC), Belin Ambrosio display-total deviation (BAD-D), anterior elevation at the thinnest point (AE-Thin), posterior elevation at the thinnest point (PE-Thin), total spherical aberrations (SA), total higher order aberrations (HOA).

**Figure 1:**
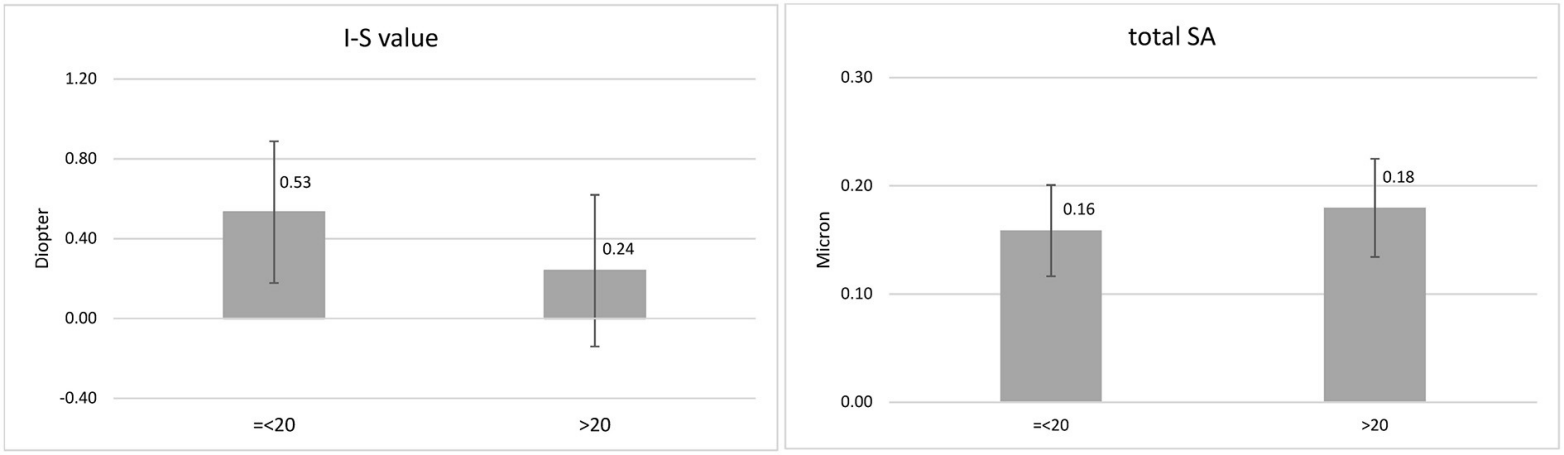
Mean and standard deviation of inferior-superior asymmetry (I-S value) and total corneal spherical aberration (SA) in the age groups ≤ 20.0 and > 20.0 years.

In the DS-N group, association of I-S value −1.05, *P* = 0.043) and total SA (12.44, *P* = 0.005) with age was significant. Figure 1 presents the mean values of these indices in the age groups ≤ 20.0 and >20.0 years. There were significant differences in ACD (*P* = 0.003), ACV (*P* = 0.005), Ksteep (*P* = 0.009), Kflat (*P* = 0.008), Kmax (*P* = 0.006), ZKmax-3 mm (*P* = 0.009), ARC (*P* = 0.026), PRC (*P* = 0.009), BAD-D(*P* = 0.003), PE-Thin (*P* = 0.050), and corneal vertical coma (*P* = 0.013) between females and males (Table 2).

**Table 2:** Gender distribution of corneal indices in Down syndrome patients with normal cornea (*n* = 154).

	**Female**	**Male**
	**Mean ±SD**	**95% CI**	**Median**	**Mean ±SD**	**95% CI**	**Median**
**ACD (mm)**	2.93 ± 0.29	2.86 to 3.00	2.93	3.09 ± 0.28	3.03 to 3.15	3.06
**ACV (mm** ^**3**^ **)**	159.88 ± 29.44	152.81 to 166.95	157.00	175.61 ± 30.88	168.95 to 182.27	168.00
**Ksteep (D)**	46.91 ± 1.75	46.49 to 47.33	46.90	46.20 ± 1.52	45.87 to 46.53	46.20
**Kflat (D)**	45.13 ± 1.55	44.76 to 45.51	45.00	44.51 ± 1.31	44.22 to 44.79	44.50
**Kmax (D)**	47.52 ± 1.77	47.09 to 47.94	47.60	46.75 ± 1.56	46.42 to 47.09	46.77
**ZKmax-3mm (D)**	46.74 ± 1.63	46.35 to 47.13	46.76	46.08 ± 1.43	45.77 to 46.39	46.08
**ARC (mm)**	7.34 ± 0.25	7.28 to 7.40	7.32	7.43 ± 0.22	7.38 to 7.48	7.41
**PRC (mm)**	6.04 ± 0.25	5.98 to 6.10	6.03	6.15 ± 0.25	6.10 to 6.20	6.18
**BAD-D**	1.98 ± 1.18	1.69 to 2.26	1.90	1.51 ± 0.83	1.33 to 1.69	1.50
**PE-Thin (μm)**	10.65 ± 8.20	8.66 to 12.63	9.50	8.96 ± 5.03	7.87 to 10.06	9.00
**Total vertical coma (μm)**	0.23 ± 0.14	0.19 to 0.26	0.22	0.18 ± 0.11	0.16 to 0.21	0.17

Bold notations: significant differences between female and male.

Anterior chamber depth (ACD), anterior chamber volume(ACV), maximum keratometry in the central 3 mm (Ksteep), minimum keratometry in the central 3 mm (Kflat), maximum keratometry in the central 8 mm (Kmax), maximum keratometry in a 3 mm zone around the steepest point (ZKmax-3mm), anterior radius of curvature centered on the thinnest point (ARC), posterior radius of curvature centered on the thinnest point (PRC), Belin Ambrosio display-total deviation (BAD-D), posterior elevation at the thinnest point (PE-Thin), total vertical coma.

## Discussion

According to the results of the preset study, the mean values of all indices were significantly different between DS-N and DS-KC groups. Comparison of corneal indices in DS patients (4–6) with non-DS populations (4–6, 9, 10) has shown that the DS patients have a thinner, shallower, steeper, more elevated (especially in the posterior part), and more aberrated corneas. In addition to the results of this study that were presented in previous reports, several studies have also pointed to differences, which casts doubts on KC diagnosis in these patients so that a range of 12.4% (2) to 71.3% (3) has been reported for KC prevalence in this population. In a very recent study using 3-D morphogeometric and volumetric analysis, Toprak et al. (11) found that DS-N patients had more aberrated corneas with a more obvious displacement of the anterior corneal apex even in the presence of a normal topograpic pattern compared to non-DS subjects, which is very important in detection of KC in these patients (12). Vega-Estrada et al. (12) also found that the posterior corneal surface of the DS subjects had lower volume and thickness and higher irregularity, steepening, and HOA compared to non-DS corneas, indicating a high degree of resemblance to mild KC. In fact, due to these similarities, it is very difficult to discriminate mild KC from the normal pattern in DS patients. In a previous report (13), we showed that among corneal indices, MCT, corneal volume, and BAD-D had a relatively good diagnostic ability in DS patients and the diagnostic power of these indices for detecting KC was not as excellent as their power in non-DS subjects. Also, HOA and coma were the best discriminators. These findings indicate the need for defining a normal range in this population.

The results of this report showed a reduction in the I-S value and an increase in SA with aging in DS-N patients aged 10-30 years. Considering the mean values of these indices in the age groups ≤ 20.0 and >20.0 years, it seems that the changes mostly occur around 20 years of age. These are normal age-related changes of the indices in DS patients, which were different from changes resulting from KC. To the best of our knowledge, there are no reports on the age-related changes of the I-S value in the normal population. It can be said a natural regularization of corneal surface occurs around the age of 20 in DS-N patients.

Amano et al. (14) evaluated a wide age group (18–69 years) of non-DS individuals and found non-significant corneal SA and significant ocular SA changes with age. Therefore, in non-DS subjects, increased ocular SA with age is a dominant phenomenon secondary to age-related changes in the anterior and posterior lens radius (15, 16). On the contrary, corneal SA increased with age in DS patients; however, since contrast sensitivity testing was not done, it was not possible to evaluate the effect of this age-related increase of SA on the vision quality. It should be specially noted that corneal aberrations have been compensated by internal optics and ocular abberations are different from corneal abberations.

In the present study, there were significant differences in almost all corneral indices except for ACT, MCT, ART-max, AE-Thin, and corneal vertical coma between male and female DS-N patients so that female had shallower, steeper, more elevated, and more aberrated corneas compared to male. Hence, sex-specific reference ranges should be considered for KC diagnosis for them. Reports from normal populations also indicate that females have steeper and more prolate corneas (17) and experience greater age-related changes of keratometry indices compared to male (18).

Several studies have shown corneal structural changes mediated by sex hormones during pregnancy and menstruation (19, 20). Sex hormones receptors, especially estrogen, increase the corneal thickness and change its curvature through water retention in physiological hormonal phases. A number of studies have investigated the effects of these hormones on KC pathogenesis. Some studies found augmented progression of the disease during pregnancy due to increased production of sex hormones (21–23). Moreover, an *in vitro* study showed that the corneal stroma houses α and β estrogen receptors and can be involved in the pathobiology of KC under the effect of estrone and estriol (24).

A limitation of this study is method of sampling and low generalizability of the results to the DS population. However, its findings can be used as a reference range for corneal indices in DS patients due to its large sample size and sampling from different sources. Another limitation of study was the small sample size of the DS-KC group and the non-homogeneous distribution of KC stages. However, the power of analysis was enough and the difference in most indices was significant between the two groups. In general, the results of the present study suggest that DS patients have a different corneal structure, even in the absence of corneal abnormalities, compared to non-DS subjects and their corneal indices are more similar to non-DS patients with mild KC; therefore, it is necessary to use defined reference ranges for detection of KC in this population. Moreover, no significant changes occur in corneal indices except for corneal symmetrization and a slight increase in SA in the age range 10–30 years. Female DS patients have shallower, steeper, more elevated, and more aberrated corneas compared to male, and therefore it is necessary to use sex-specific normative values for detection of corneal abnormalities in this population.

## Data availability statement

The raw data supporting the conclusions of this article will be made available by the authors, without undue reservation.

## Ethics statement

The Studies involving human participants were reviewed and approved by Ethics Committee of Tehran University of Medical Sciences (ID: 1397.091). Written informed consent to participate in this study was provided by the participants' legal guardian/next of kin.

## Author contributions

HH: concept and design of study, interpretation and data, and critical revision of manuscript. SA: analysis of data, and writing the manuscript. Both authors contributed to the article and approved the submitted version.

## Conflict of interest

The authors declare that the research was conducted in the absence of any commercial or financial relationships that could be construed as a potential conflict of interest.

## Publisher's note

All claims expressed in this article are solely those of the authors and do not necessarily represent those of their affiliated organizations, or those of the publisher, the editors and the reviewers. Any product that may be evaluated in this article, or claim that may be made by its manufacturer, is not guaranteed or endorsed by the publisher.
